# Enhanced sensitivity and scalability with a Chip-Tip workflow enables deep single-cell proteomics

**DOI:** 10.1038/s41592-024-02558-2

**Published:** 2025-01-16

**Authors:** Zilu Ye, Pierre Sabatier, Leander van der Hoeven, Maico Y. Lechner, Teeradon Phlairaharn, Ulises H. Guzman, Zhen Liu, Haoran Huang, Min Huang, Xiangjun Li, David Hartlmayr, Fabiana Izaguirre, Anjali Seth, Hiren J. Joshi, Sergey Rodin, Karl-Henrik Grinnemo, Ole B. Hørning, Dorte B. Bekker-Jensen, Nicolai Bache, Jesper V. Olsen

**Affiliations:** 1 State Key Laboratory of Common Mechanism Research for Major Diseases, Suzhou Institute of Systems Medicine, Chinese Academy of Medical Sciences & Peking Union Medical College, Suzhou, China; 2 Novo Nordisk Foundation Center for Protein Research, University of Copenhagen, Copenhagen, Denmark; 3 Cardio-Thoracic Translational Medicine Laboratory, Department of Surgical Sciences, Uppsala University, Uppsala, Sweden; 4 Thermo Fisher Scientific (China) Co. Ltd, Shanghai, China; 5 Cellenion SASU, Lyon, France; 6 Copenhagen Center for Glycomics, Department of Cellular and Molecular Medicine, University of Copenhagen, Copenhagen, Denmark; 7 Evosep Biosystems, Odense, Denmark

**Keywords:** Proteomic analysis, Mass spectrometry

## Abstract

Single-cell proteomics (SCP) promises to revolutionize biomedicine by providing an unparalleled view of the proteome in individual cells. Here, we present a high-sensitivity SCP workflow named Chip-Tip, identifying >5,000 proteins in individual HeLa cells. It also facilitated direct detection of post-translational modifications in single cells, making the need for specific post-translational modification-enrichment unnecessary. Our study demonstrates the feasibility of processing up to 120 label-free SCP samples per day. An optimized tissue dissociation buffer enabled effective single-cell disaggregation of drug-treated cancer cell spheroids, refining overall SCP analysis. Analyzing nondirected human-induced pluripotent stem cell differentiation, we consistently quantified stem cell markers OCT4 and SOX2 in human-induced pluripotent stem cells and lineage markers such as GATA4 (endoderm), HAND1 (mesoderm) and MAP2 (ectoderm) in different embryoid body cells. Our workflow sets a benchmark in SCP for sensitivity and throughput, with broad applications in basic biology and biomedicine for identification of cell type-specific markers and therapeutic targets.

## Abstract (web summary)

Chip-Tip is a label-free quantification-based single-cell proteomics workflow for deep single-cell proteomics, which identifies over 5,000 proteins and 40,000 peptides in single HeLa cells.

## Main

Multicellular organisms are composed of specialized tissues that consist of various cell types, each performing distinct functions. The unique properties of each cell type arise from the interaction of their genetic information with internal factors. Studying individual cells is critical since cells can exhibit diverse behaviors under identical external environments^1^. Single-cell RNA sequencing has transformed cell biology by providing a granular view of gene expression patterns, spatial cell architecture, cellular heterogeneity and dynamic cellular responses^2,3^. However, messenger RNAs (mRNAs) serve as an intermediate step in gene expression and do not directly reflect cellular activity. Studying proteins provides a more direct and comprehensive understanding of cellular functions, regulatory mechanisms and disease processes compared to studying mRNA changes alone. Protein analysis captures post-translational modifications (PTMs), protein diversity and functional aspects that are not reflected in mRNA analyses.

Single-cell proteomics (SCP) is emerging as the next frontier in proteomics and has already enhanced our understanding of cellular differentiation and diseases by allowing for the direct measurement of single-cell proteomes and their PTMs^4–9^. This capability is instrumental in delineating the functional phenotypes within cell populations, elucidating cellular and embryonic development, predicting disease trajectories and pinpointing specific surface markers and potential therapeutic targets unique to each cell type^10,11^.

However, SCP is nascent and faces challenges including limited sequence depth, throughput and reproducibility, constraining its broader utility. This study introduces key methodological advances, which considerably improve the sensitivity, coverage and dependability of protein identification from single cells. SCP predominantly uses two main quantitative techniques: label-free^6,12,13^ or multiplexed^7,8,14^ analysis. Label-free quantification (LFQ) analysis presents the simplest SCP workflow by lysing cells to extract proteins, which are then digested into peptides, separated via liquid chromatography (LC) and analyzed using mass spectrometry (MS), thus identifying and quantifying peptides from single cells. In a pioneering study, Li et al. developed a nanoliter-scale oil-air-droplet chip^9^ and achieved increased analytical sensitivity for SCP using label-free shotgun proteomics. Multiplexed analysis, on the other hand, uses isobaric labeling, allowing for the simultaneous analysis of multiple samples by chemically tagging peptides with unique stable isotope encoded mass tags. The recent innovation of nonisobaric multiplexed data-independent acquisition (plexDIA^15^ or mDIA^16^) has combined the strengths of DIA with multiplexing, improving protein quantification rates and accuracy without the ratio compression problems associated with tandem mass tags^17–19^. State-of-the-art SCP, identifying around 1,000–2,000 protein groups per cell and 1,500–2,500 proteins across cells, required improvements in MS sensitivity and sample preparation^12^. Yet, the loss of peptides during sample preparation and analysis due to protein adsorption loss, chemical modifications and ion manipulation remains a challenge^20^.

**Fig. 1:**
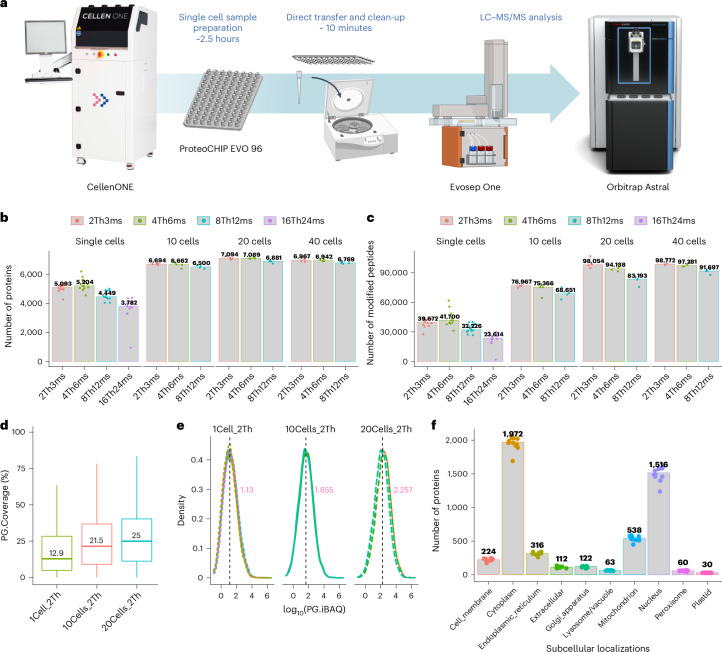
Workflow and results of Chip-Tip SCP. **a**, Schematic of the Chip-Tip workflow. **b**, Number of proteins identified in single, 10, 20 and 40 HeLa cells using variable nDIA settings. **c**, Number of peptides identified in single, 10, 20 and 40 HeLa cells using variable nDIA settings. **d**, Protein sequence coverage percentages for single-, 10- and 20-cell samples. For the boxplots, the lower and upper hinges correspond to the first and third quartiles. The upper whisker extends from the hinge to the largest value no further than 1.5× IQR from the hinge (where IQR is the inter-quartile range). The lower whisker extends from the hinge to the smallest value at most 1.5× IQR of the hinge. **e**, Distribution of iBAQ values showing quantification range and consistency across different cell counts. **f**, Overview of protein localization across cellular compartments. Spectronaut (v.18) was used for the search. Panel **a** was created with BioRender.com. Source data

To address these challenges, we developed a nearly lossless LFQ-based SCP method, Chip-Tip, which identifies over 5,000 proteins and 40,000 peptides in single HeLa cells. Our workflow involves single cell dispensing and sample preparation using the cellenONE with a proteoCHIP EVO 96 and direct transfer to Evotip disposal trap columns, and subsequent analysis using the Evosep One LC with Whisper flow gradients coupled to narrow-window DIA (nDIA) on the Orbitrap Astral mass spectrometer^21^ (Fig. 1a). Our study includes a systematic evaluation of database search tools and an error-rate estimation using an entrapment approach, ensuring reliable data analysis. Furthermore, our method has enabled the direct investigation of PTMs in single-cell proteomes, achieving deep coverage in phosphorylation and glycosylation without previous enrichment. The application of this technique to spheroid samples with a new dissociation buffer underscores its robustness, offering important insights into the proteomic intricacies of individual cells and their implications for biological processes and disease states. Finally, we studied undirected differentiation of human-induced pluripotent stem cells (hiPSCs) into multiple cell types through embryoid body (EB) induction. We identified up to 4,700 proteins in hiPSCs and 6,200 in cells from EBs and quantified low-abundant stem cell transcription factors in hiPSCs and different cell lineage markers in EBs.

## Results

### Roadmap to deep and high-throughput LFQ-SCP using Chip-Tip

**Extended Data Fig. 1:**
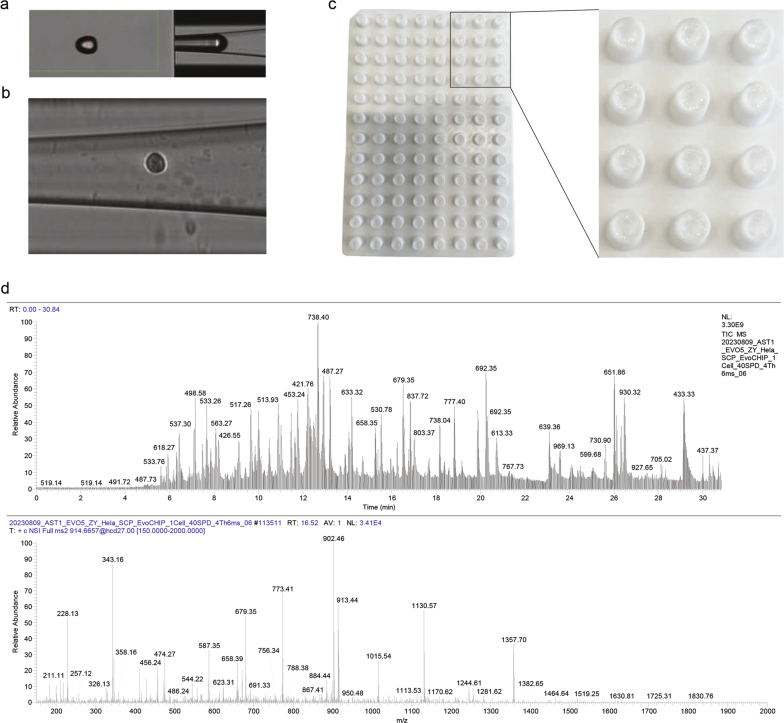
Examples of each step in the SCP workflow. **a**) Example of a droplet in cellenONE. This is used in buffer dispensing. **b**) Example of an isolated HeLa cell in cellenONE. **c**) Picture of a proteoCHIP EVO 96. This is the picture taken after the sample transfer from the chip to Evotips. **d**) Example of a total ion current (TIC) and an MS/MS spectrum in a single cell sample.

**Extended Data Fig. 2:**
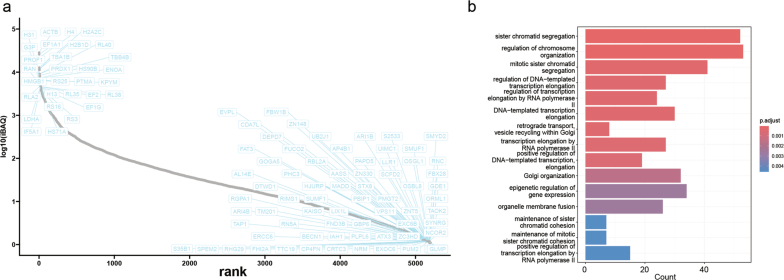
iBAQ distribution of the single cell proteome. **a**) Distribution of the iBAQ values a single cell sample, with labels indicating some of the highest and lowest abundant proteins. **b**) Gene ontology enrichment analysis specifically focusing on the 1500 proteins with the lowest abundance. P values were calculated using a one-sided Fisher’s Exact Test and subsequently adjusted using the Benjamini-Hochberg (BH) correction method.

Key considerations for SCP sample preparation workflows include minimizing surface adsorption losses by keeping samples as concentrated as possible and reducing buffer evaporation and pipetting steps for reproducibility and maximizing detection sensitivity. Consequently, we focused on developing a SCP workflow characterized by ultra-high sensitivity (Fig. 1a and Supplementary Table 1). Initially, cells were isolated and processed using a one-pot technique in the cellenONE X1 platform (Extended Data Fig. 1a,b). A key innovation is the proteoCHIP EVO 96, designed for single-cell sample preparation, which operates with minimized volumes at the nanoliter level, enabling the simultaneous proteomics sample preparation of up to 96 cells in parallel (Extended Data Fig. 1c). This chip is precisely tailored for compatibility with the Evosep One LC system for a streamlined sample transfer process that is free from additional pipetting steps. For analysis by LC with tandem MS (LC–MS/MS), we combined the Whisper flow methods on Evosep One LC with high-precision IonOpticks nanoUHPLC columns to maximize sensitivity and chromatographic performance (Extended Data Fig. 1d). Our recently introduced nDIA method^21^, applied on the Orbitrap Astral mass spectrometer, greatly amplifies the sensitivity and efficiency of the SCP analysis. To optimize the nDIA approach for the Orbitrap Astral^22,23^, which had not been previously used for label-free single-cell analysis, we initially assessed different nDIA methods, examining different quadrupole isolation windows and scaled ion injection times for DIA–MS/MS scans, accordingly. Through this comparative analysis, we determined the optimal nDIA method and found that using 4-Th DIA windows and 6-ms maximum injection time (4Th6ms) resulted in the highest proteome coverage, leading to the identification of a median number of 5204 proteins in single HeLa cells and >6,000 proteins in one of the HeLa cell preparations (Fig. 1b). We observed that as the injection time increased in the 8Th12ms and 16Th24ms methods, there was a corresponding decline in the number of proteins identified, likely attributable to an increase in chemical noise signals within the analyzer. When we expanded our method to process larger cell quantities, we identified more than 7000 proteins from a batch of only 20 cells. The achieved depth at the peptide level was particularly remarkable, with median identifications of 41,700 peptides for single-cell samples and 98,054 peptides for samples from 20 cells (Fig. 1c), resulting in median protein sequence coverage of 12.9% for single cells and 25% for 20-cell samples (Fig. 1d). This profound peptide coverage facilitated highly accurate protein quantification, with intensity-based absolute quantitation (iBAQ) values exhibiting an extensive dynamic range spanning several orders of magnitude (Fig. 1e and Extended Data Fig. 2). The relative protein abundance estimates also demonstrated an almost linear relationship of iBAQ values across samples with varying cell numbers (Fig. 1e). Our comprehensive SCP profiles included proteins from all subcellular localizations^24^, with a notable identification of over 200 proteins on plasma membranes, emphasizing the robustness of the method (Fig. 1f).

### Exploring the carrier proteome effect in label-free SCP

**Fig. 2:**
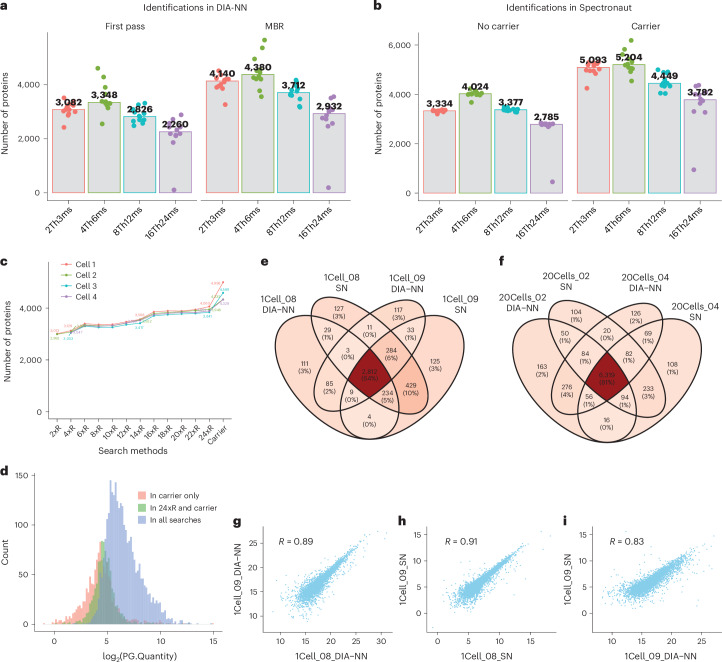
Evaluating the carrier proteome effect in SCP. **a**, Protein identifications in single-cell samples using DIA-NN with and without MBR feature, compared across various nDIA methods. **b**, Protein identifications in single-cell samples with and without carrier proteomes in Spectronaut. **c**, Trend analysis showing the increase in protein identifications when single-cell data is searched with a growing number of single-cell files and with carrier proteomes in Spectronaut. **d**, Histogram illustrating the distribution of protein abundances identified across different search strategies, highlighting the advantage of carrier proteomes in detecting low-abundance proteins. **e**, Venn diagram comparing protein identifications in two single-cell samples in DIA-NN and Spectronaut. **f**, Venn diagram of protein identifications comparing two 20-cell samples using DIA-NN and Spectronaut. **g**, Scatter plot displaying the quantification correlation of proteins identified between two single-cell runs in DIA-NN. **h**, Scatter plot displaying the quantification correlation of proteins identified between two single-cell runs in Spectronaut. **i**, Scatter plot presenting the quantification correlation between Spectronaut and DIA-NN for the same single-cell sample, with correlation coefficients indicating the degree of agreement. In **e** and **g**–**i**, 08 and 09 are two different HeLa cells of similar sizes. Source data

**Extended Data Fig. 3:**
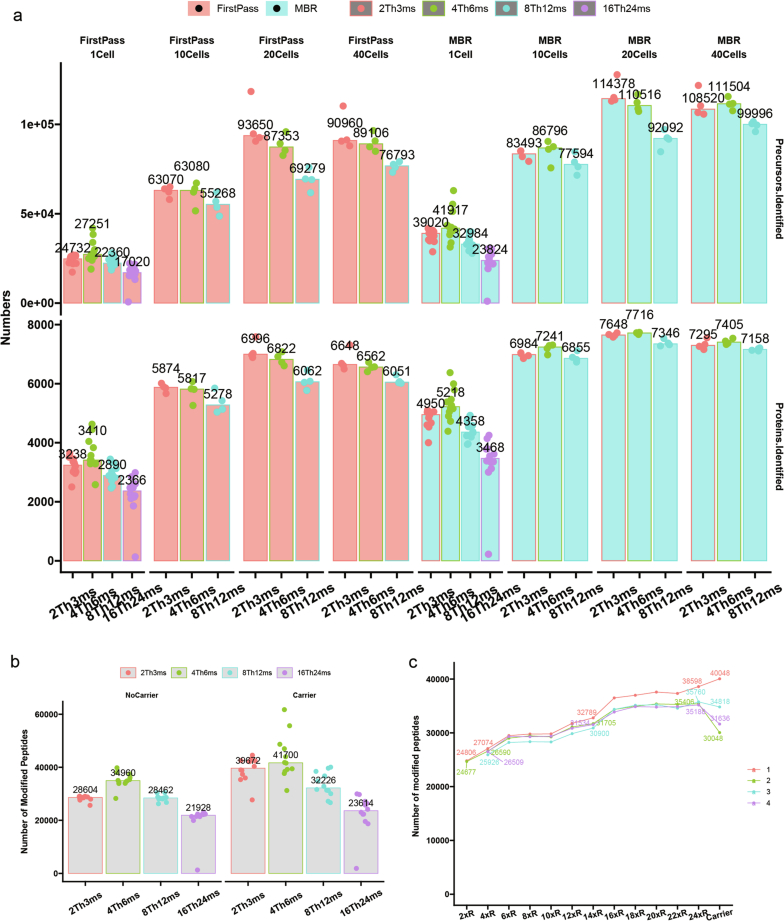
Carrier proteome effect in label-free single cell proteomics. **a**) Number of proteins and precursors identified in the HeLa samples in DIA-NN. **b**) Number of modified peptides identified in the single cell HeLa samples in Spectronaut. **c**) Trend analysis showing the numbers in peptide identifications when single-cell data is searched with different number of single-cell files and with carrier proteomes in Spectronaut.

In LFQ-based SCP, each cell is analyzed independently by LC–MS/MS with nDIA, free from the signal interferences characteristic of multiplexed SCP. However, the proteomic profiles identified from single cells can be largely influenced by the strategy employed for the spectra-to-database matching with a peptide search engine. Two strategies are currently employed to enhance identification numbers using two popular DIA database search tools, Spectronaut^25^ and DIA-NN^26^. In Spectronaut, this is the directDIA+ or spectral library-free based approach, which includes searching alongside matched samples of higher quantities (such as 1-ng digests or those from 20 cells), whereas in DIA-NN it is the match-between-run (MBR)^27^ feature, similarly paired with higher quantity samples. While these search strategies differ between tools, both can elevate identification numbers in single-cell samples by incorporating data from matched higher quantity samples. These methods are widely applied in SCP, yet their precise effects remain to be fully understood. We performed a systematic evaluation to elucidate the effect of using such a ‘carrier proteome’ for SCP by benchmarking between Spectronaut and DIA-NN, alongside their respective search strategies. When compared to searches with only single-cell samples, inclusion of carrier proteomes resulted in a substantial increase in the number of identifications (Figs. 2a,b and Extended Data Fig. 3a,b). Further investigation into the carrier proteome effect in Spectronaut revealed that as the number of single-cell files in a search increased, the number of identifications also rose incrementally. When carrier proteomes were included, there was a marked enhancement in identifications, increasing from around 4,000 to approximately 5,000 (Fig. 2c and Extended Data Fig. 3c). Analysis of the abundance of proteins identified through different search strategies indicated that searches incorporating more cells could discern proteins of lower abundance, with the carrier proteome enabling the identification of the least abundant proteins (Fig. 2d). To compare the efficacy of these search strategies, we examined two separate runs from single-cell samples (Fig. 2e) and from 20-cell samples (Fig. 2f), contrasting their outcomes in DIA-NN and Spectronaut. The results showed a big overlap in identifications, suggesting consistency across different cells and search tools. However, the protein quantification correlation between the two cells was marginally higher in Spectronaut with *R* = 0.91 compared to DIA-NN with *R* = 0.89 (Fig. 2g,h). Conversely, the correlation between Spectronaut and DIA-NN for the same cell was not as high with *R* = 0.83 (Fig. 2i).

**Extended Data Fig. 4:**
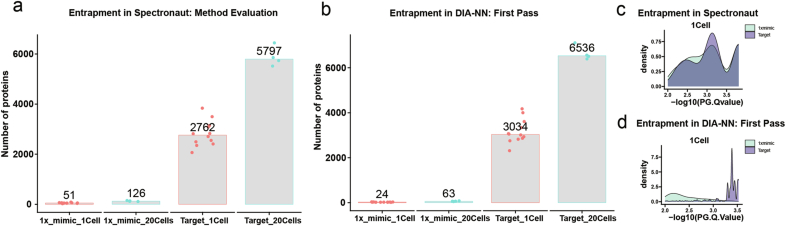
Error rate estimation in single cell proteomics through entrapment analysis. (**a-b**) Number of proteins identified in SCP using a 1x mimic database using Spectronaut (**a**) and DIA-NN (**b**). (**c-d**) Density plot for the distribution of PG.Qvalues of false and target identifications in single-cell samples using Spectronaut (**c**) and DIA-NN (**d**). Note, raw files were searched in Spectronaut with Method Evaluation option checked, and the first pass results were used from DIA-NN.

The ultra-low abundance of single-cell proteomes raises questions about the confidence in peptide and protein identifications, particularly with the increased numbers achieved using the Orbitrap Astral mass spectrometer. To address these concerns, we conducted a false discovery rate (FDR) benchmark by using an entrapment database search strategy with both Spectronaut and DIA-NN to empirically estimate the error rate of identifications^28^. We initiated this by conducting an entrapment experiment with a 1× larger shuffled human mimic protein database, analyzing single-cell samples in combination with 20-cell samples serving as a carrier proteome with default FDR parameter settings of 0.01 at protein level. The resulting empirical FDR at the protein level, corrected for the mimic database size, was estimated at approximately 3% in Spectronaut and 1% in DIA-NN (Extended Data Fig. 4).

### Faster LC methods and FAIMS further enhance SCP performance

**Fig. 3:**
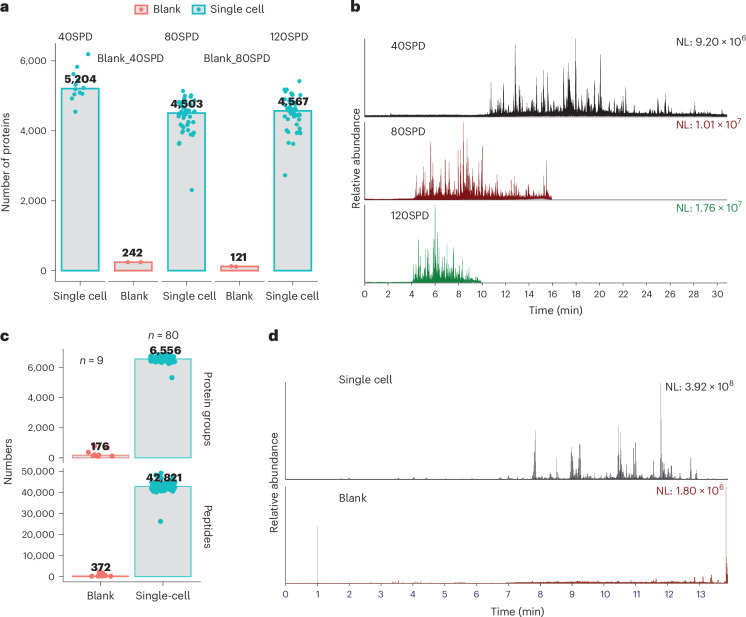
Faster LC methods and FAIMS further enhance SCP performance. **a**, Protein identifications in single-cell samples and blank samples using 40SPD, 80SPD and 120SPD Whisper Zoom methods in Evosep One LC. **b**, Representative total ion current at MS2 level of single-cell samples using 40SPD, 80SPD and 120SPD methods in Evosep One. **c**, Protein identifications in single-cell samples and blank samples using Vanquish Neo with FAIMS Pro Duo interface. **d**, Representative total ion chromatogram (TIC) at MS2 level of a single cell and a blank sample. In **c** and **d**, individual HeLa cell samples were prepared in a 96-well plate and directly injected into an Orbitrap Astral mass spectrometer coupled with FAIMS Pro Duo interface and Vanquish Neo LC system. Samples were analyzed in a 14-min gradient method. Source data

Recognizing the throughput limitation in MS as a major bottleneck for label-free SCP, our methodology has innovated a solution that increases the throughput from 40 samples per day (SPD) to 80SPD and even 120SPD on Evosep One LC system with the Whisper Zoom method. This optimized LC method not only improves throughput but also enhances chromatographic performance and maintains a high level of sensitivity, identifying >4,500 proteins in individual HeLa cells using 80SPD and 120SPD methods (Fig. 3a,b). It should also be noted that the 80SPD and 120SPD methods use a 5 cm analytical LC column, while the 40SPD method makes use of a 15 cm column, which contributes to the slight difference in performance. Furthermore, we tested for potential contamination and carryover throughout the workflow by injecting samples prepared without a single cell. These blank samples, containing only Master Mix buffer and prepared identically to real single-cell samples, resulted in very few protein identifications when analyzed alongside real single-cell samples (Fig. 3a), demonstrating the robustness and low contamination risk of our workflow.

Next, we evaluated another setup using the Vanquish Neo LC and Orbitrap Astral equipped with a front-end high field asymmetric waveform ion mobility spectrometry (FAIMS) interface to minimize background interference. Single cells were isolated using cellenONE and prepared in a low-bind 96-well plate for direct MS sample injection. Using a single compensation voltage of −48 V, the FAIMS Pro Duo interface further enhanced analytical performance, resulting in the identification of over 6,500 proteins in single HeLa cells (Fig. 3c). A key consideration with this well-plate format preparation and direct injection into the LC–MS system is the absence of a sample cleanup step, which could potentially affect the cleanliness, robustness and longevity of the LC–MS systems. Despite this, blank samples from this workflow also demonstrated contamination-free performance (Fig. 3c,d).

### In-depth PTM analysis without enrichment in SCP

**Fig. 4:**
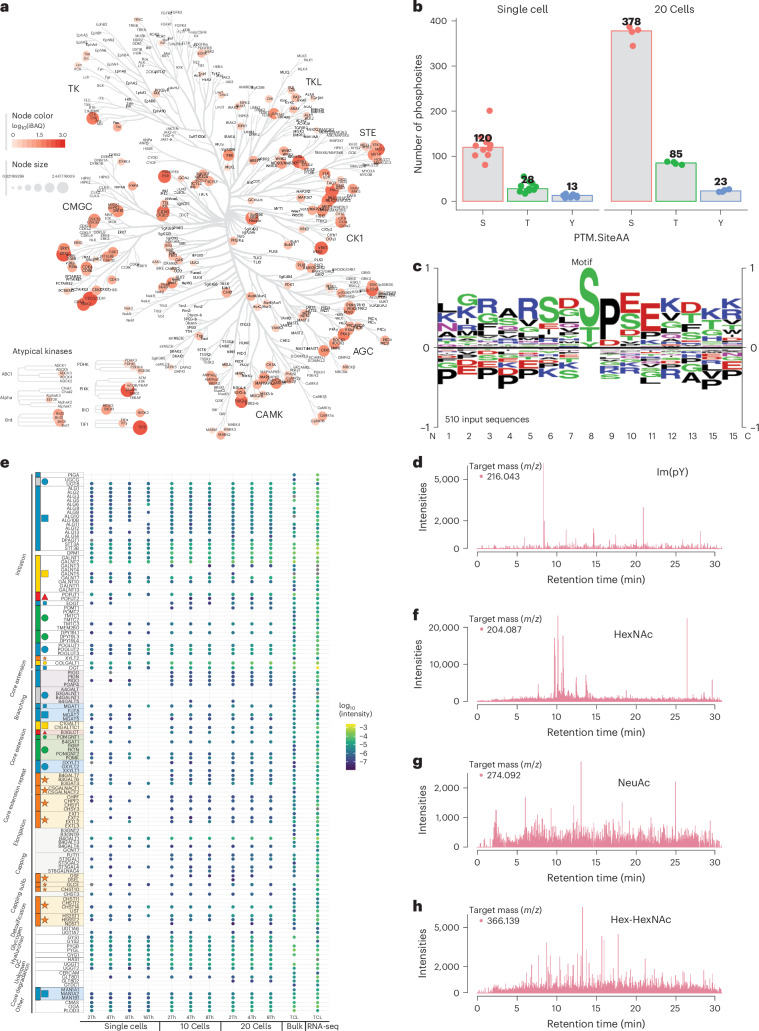
PTM profiling in SCP. **a**, Kinome tree representation showing the quantification of 168 kinases across major kinase families in single cells, with node color and size indicating iBAQ abundances. **b**, Bar graph depicting the number of phosphosites identified for serine (S), threonine (T) and tyrosine (Y) residues in single- and 20-cell samples. **c**, Sequence logo analysis illustrating the most common phosphorylation motifs identified in single-cell samples. **d**, XIC of immonium ion for phosphor-Tyr (*m/z* 216.0426) across the LC elution profile. **e**, Visualization of the glycogenes identified in single cells. **f**, XIC of oxonium ion for HexNAc (*m/z* 204.087) indicating glycan presence in the MS2 spectra. **g**, XIC of oxonium ion for NeuAc (*m/z* 274.092) reflecting glycan abundance in the MS2 spectra. **h**, XIC of oxonium ion for the disaccharide Hex-HexNAc (*m/z* 366.139) reflecting glycan abundance in the MS2 spectra. Source data

**Extended Data Fig. 5:**
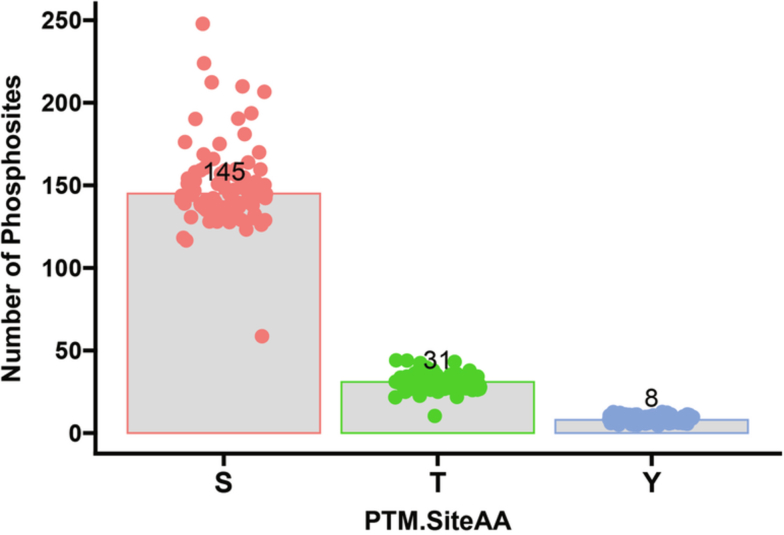
Number of phosphosites identified using the Vanquish Neo and FAIMS approach. Bar and dot plots showing the number of phosphosites on different residues identified in single HeLa cell samples. The single cell samples were prepared and analyzed using the Vanquish Neo and FAIMS approach.

**Extended Data Fig. 6:**
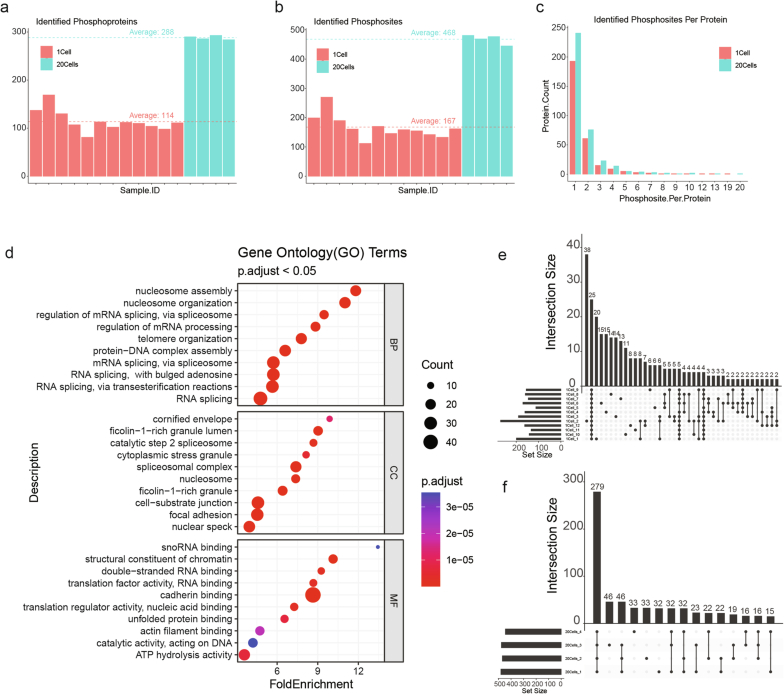
Analysis of the phosphosites using Chip-Tip method. (**a-b**) Identifications of phosphoproteins (**a**) and phosphosites (**b**) in the single cell and 20-cell samples. (**c**) Identified phosphosites per protein in single cell samples. (**d**) GO enrichment analysis of the phosphoproteins in the single cell samples. (**e-f**) UpSet plot of the identified phosphosites in single cell (**e**) and 20-cell (**f**) samples.

The exceptional depth achieved at the peptide precursor level in our SCP datasets not only enhances protein identification and quantification but could also unveil a wealth of information pertaining to PTMs. Due to the substoichiometric nature of PTMs, global analysis of any PTM by LC–MS/MS usually requires specific enrichment of the PTM-bearing peptides before MS analysis. However, with the high peptide coverage achieved in SCP, we speculated that identification of PTMs without specific enrichment could be possible. To test this hypothesis, we delved into the prevalence of two key PTMs of high cellular importance, phosphorylation and glycosylation, within the single-cell proteome samples, bypassing any specific enrichment processes. Focusing on the enzymes catalyzing the transfer of phosphate groups from ATP to target proteins, we quantified 168 protein kinases within single cells, encompassing all principal kinase families (Fig. 4a). Notably, kinases such as CDK1 from the CMGC group and MAPK1 from the STE group exhibited the highest abundances, whereas tyrosine kinases were less abundant. Subsequently, we conducted a database search for serine, threonine and tyrosine phosphorylation as a variable modification in single-cell samples, using 20-cell samples as a carrier proteome. Although we did not do any specific cell perturbation or sample treatment to preserve the phosphorylation sites, the search approach led to the confident identification of a median of 120 phospho-Ser, 28 phospho-Thr and 13 phospho-Tyr sites in single cells, with high site localization probabilities (Fig. 4b and Extended Data Fig. 5). An average of 114 proteins were identified as phosphoproteins and they are mostly involved in nucleosome assembly and organization (Extended Data Fig. 6). A sequence logo analysis highlighted prevalent phosphorylation motifs such as the proline-directed SP motif corresponding to substrates for abundant kinases such as CDKs, aligning closely with our kinome data (Fig. 4c). An extracted ion chromatogram (XIC) screening of DIA–MS/MS spectra for the selective mass-deficient immonium ions for phospho-Tyr^29^ at *m/z* 216.042 showed intensive signals across the entire LC elution profile (Fig. 4d). We also investigated protein glycosylation patterns and detected multiple glycosyltransferases across all glycosylation pathways (Fig. 4e). Using a strategy akin to the phospho-Tyr immonium ion screening, we performed oxonium ion screening^30,31^ for common glycans, including the monosaccharides HexNAc (Fig. 4f), NeuAc (Fig. 4g) and the disaccharide Hex-HexNAc (Fig. 4h). All glycans appeared abundantly and were present in most MS2 spectra, even when using nDIA. The screening of both immonium and oxonium ions indicated a pervasive presence of PTMs, such as protein phosphorylation and glycosylation, in single cells. Nevertheless, the precise identification of the modified peptides remains challenging due to current limitations in database search algorithms.

### SCP analysis of spheroid post-5-FU exposure

**Fig. 5:**
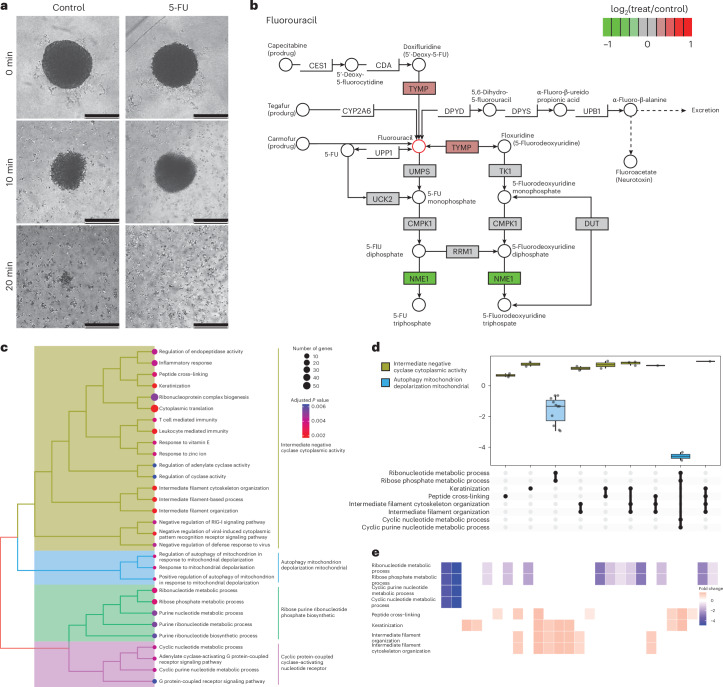
Analysis of the 5-FU impact on spheroid cells using Chip-Tip. **a**, Comparative images illustrating the morphological changes in spheroids untreated and treated with 5-FU, showing increased disintegration and cell detachment in treated spheroids over time. The experiment was performed once. Scale bars, 500 μm. **b**, Metabolic pathway diagram of 5-FU indicating the downregulation of NME1 and upregulation of TYMP, proteins integral to the metabolism and activation of 5-FU. **c**, Hierarchical clustering of GO terms related to cellular processes affected by 5-FU treatment, highlighting the involvement of cyclase cytoplasmic activity and ribose purine synthesis in its action mechanism. **d**, UpSet plot of the top GO terms, and its associated grouped mechanisms, post-5-FU treatment, detailing the upregulation and downregulation of biological processes associated with 5-FU treatment. The horizontal line in the boxplots represent the median, 25th and 75th percentiles and whiskers represent measurements to the 5th and 95th percentiles. **e**, Heatmap of protein alterations across identified GO terms.

To demonstrate the applicability of our SCP method with 80SPD, we next intended to capture the transformative effects of the chemotherapeutic drug 5-fluorouracil (5-FU) on colorectal cancer cells grown as a spheroid. The spheroids subjected to 5-FU demonstrate increased disintegration over time, after treatment with a recently developed disaggregation buffer for 20 minutes, whereas control spheroids retained their compact structure, indicative of the impact of 5-FU on cell cohesion (Fig. 5a). After single-cell analysis with 80SPD methods, we identified >2,500 proteins in total. The activation pathway of 5-FU reveals a slight upregulation of TYMP, crucial for converting 5-FU into its DNA-incorporating active form; these changes are crucial indicators of drug efficacy (Fig. 5b). The hierarchical clustering of gene ontology (GO) terms underscores the involvement of biological processes such as cyclase cytoplasmic activity and ribose purine synthesis, which are directly affected by mechanism of 5-FU action on ribosomal RNA (rRNA) synthesis, with ribosome and purine and/or pyrimidine synthesis being the main target of 5-FU (Fig. 5c). The representation of these pathways aligns with the known impact of 5-FU on the nucleotide synthesis pathways. An UpSet plot of the altered GO terms further dissects the effects of 5-FU, detailing which processes are most affected by the treatment and their regulatory trends (Fig. 5d). The alteration in proteins associated with these GO terms highlights specific proteins such as ADCY, which contributes to pyrophosphate formation, and keratin, integral for spheroid structural integrity and changes in the filaments organization in the stages of apoptosis. These findings suggest a targeted disruption by 5-FU on spheroid stability and purine metabolism, a reflection of the ability of the drug to interfere with key cellular functions (Fig. 5e).

### hiPSC differentiation

**Fig. 6:**
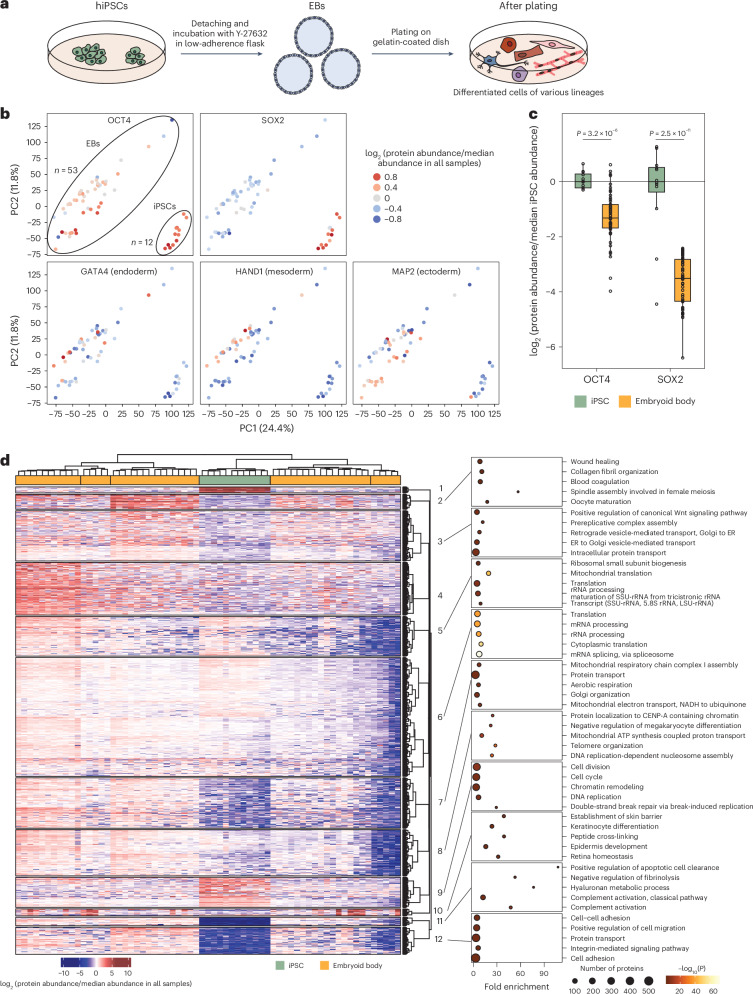
Single-cell analysis of EB induction from hiPSCs. **a**, EB induction from hiPSCs. **b**, PCA of normalized protein abundances in hiPSCs and EB cells. Points corresponding to single cells are colored according to the abundance of specific markers (OCT4, SOX2, GATA4, HAND1 and MAP2) normalized to the median abundance in all samples. *n* = 12 for hiPSCs and *n* = 50 for EB cells. **c**, Abundance of OCT4 and SOX2 divided by the median abundance in hiPSCs. The horizontal line in the boxplots represent the median, 25th and 75th percentiles and whiskers represent measurements to the 5th and 95th percentiles. The *P* values were calculated using a two-sided Student *t*-test and no adjustments were made for multiple comparisons. *P* < 0.05 was considered as significant. *n* = 12 for hiPSCs and *n* = 53 for EB cells. **d**, Unsupervised hierarchical clustering using canberra and ward.D2 methods of proteins that shows significant regulation (*P* < 0.05 from ANOVA) between cell clusters (1–6) (left). GO biological processes enrichment analysis of the protein clusters from the heatmap (right). *n* = 12 for hiPSCs and *n* = 53 for EB cells. ER, endoplasmic reticulum. Source data

**Extended Data Fig. 7:**
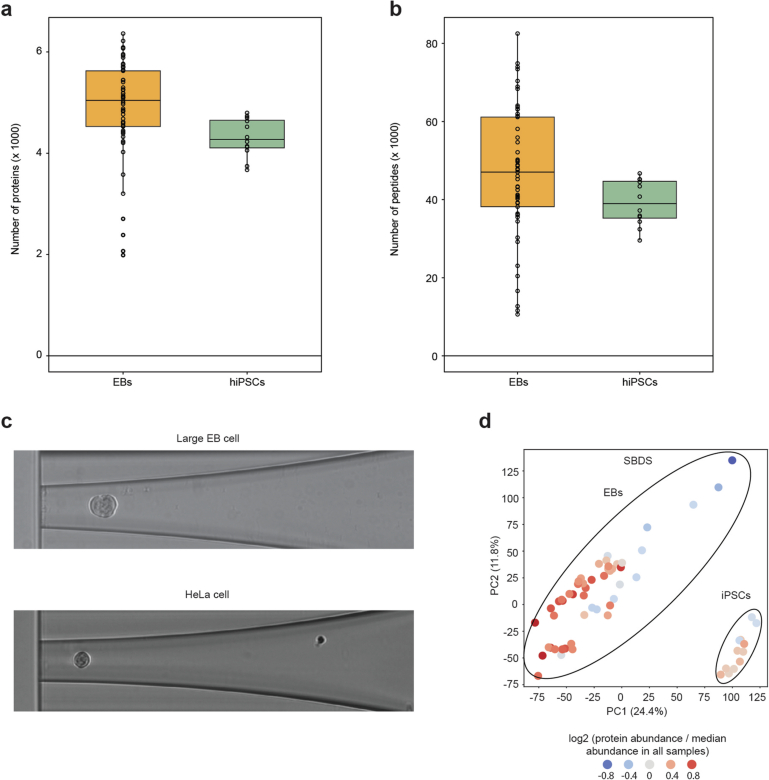
Number of proteins and peptides quantified in single cells from EBs and hiPSCs and changes in SBDS abundance. **a**) Number of proteins. **b**) Number of peptides. The horizontal line in the boxplots represent the median, 25th and 75th percentiles and whiskers represent measurements to the 5th and 95th percentiles. n = 12 for hiPSCs and n = 50 for EB cells. **c**) Example of a large EB cell (top) compared to a HeLa cell (bottom) before dispensing with cellenONE. **d**) Principal component analysis of normalized protein abundances in hiPSCs and EB cells. Points corresponding to single cells are colored according to the abundance of SBDS protein normalized to the median abundance in all samples as in Fig. 6b. Source data

Since our workflow allow us to quantify more than 5,000 proteins in single HeLa cells, this analytical depth is sufficient to obtain information on specific cell markers. Thus, we next studied undirected differentiation of hiPSCs into multiple cell types through EB induction, mimicking the characteristics of early-stage embryos. The challenges in stem cell research by SCP are that certain embryonic transcription factors such as OCT4 and NANOG are translated at a very low copy number but are fundamental to cellular identity for hiPSCs, so analytical depth is paramount. This is also the case for cell-specific lineage markers, which start appearing on hiPSCs differentiation and are sometimes only present in a handful of cells in the population. We quantified up to 4,700 proteins in hiPSCs and 6,200 in large cells from EBs (Supplementary Table 2 and Extended Data Fig. 7a–c). Principal component analysis (PCA) showed a clear separation between hiPSCs and EBs, and the distance between some EB cells was on par with the distance between EBs and hiPSCs suggesting that EB cells can greatly differ from each other (Fig. 6b). We consistently quantified OCT4 in hiPSCs and in some of the EB cells demonstrating that we can indeed detect sparsely expressed transcription factors even in single cells. We detected SOX2, another stem cell marker that we found to be highly expressed in hiPSCs, and also detected multiple lineages markers such as GATA4 (endoderm), HAND1 (mesoderm) and MAP2 (ectoderm). These markers were more expressed in EB cells but with great variability, consistent with the fact that these cells differentiate into the three germ layers (Fig. 6b). We also observed early increase in SBDS protein abundance in most EB cells on differentiation (Extended Data Fig. 7d), which is a feature of pluripotent stem cells early differentiation^32^. Finally, OCT4 and SOX2 showed significantly higher abundance in hiPSCs compared to EB cells, as expected (Fig. 6c).

To study global differences between EBs and iPSCs and between different EB cells, we performed unsupervised hierarchical clustering that highlighted six different cell clusters with one of those being only composed of iPSCs, in line with the separation observed by the PCA. Next, we performed statistical analysis using analysis of variance (ANOVA) between cell clusters and performed unsupervised hierarchical clustering of proteins that showed significant differences (*P* < 0.05) (Fig. 6d). Finally, we performed GO term enrichment on the protein clusters highlighted by the analysis. Various cell functions are enriched highlighting physiological differences between each cell clusters such as difference in Wnt signaling, respiration, cell cycle, DNA replication, mRNA and rRNA processing, mitochondrial function, cell adhesion and migration. Human pluripotent stem cells differ from most cells on several biological features namely: metabolism as they mostly use glycolysis even in presence of oxygen while differentiated cells usually employ oxidative phosphorylation^33,34^; cell cycle as their cell cycle is shortened and they divide faster than most other cell types^35^; chromatin state as they maintain their chromatin in an open state^36^; adhesion to the extracellular matrix where they require specific proteins to adhere, migrate and maintain their pluripotent state such as laminin 521 (ref. ^37^). These features were also displayed in the GO enrichment highlighting lower level of proteins involved in aerobic respiration and mitochondrial respiratory chain complex I assembly (cluster 7), lower level of adherence proteins (cluster 12), but higher levels of proteins involved in cell cycle, DNA replication and chromatin remodeling in hiPSCs compared to EB cells (cluster 9). Therefore, our analysis can deliver valuable biological insight into stem cell differentiation both in term of general pathways but also in term of specific, low-abundance biomarkers.

## Discussion

Our study marks a big leap in the field of SCP, achieving a huge enhancement in sensitivity, with identifications increasing from approximately 2,000 proteins to 5,000–6,500 proteins. This achievement underscores the evolution of the powerful proteomics technologies empowering researchers to delve deeper into the molecular intricacies of individual cells. The combination of near lossless single-cell sample preparation using the cellenONE with the ultra-high sensitivity provided by the Evosep One Whisper flow gradient and nDIA on the Orbitrap Astral is a powerful setup enabling SCP with high coverage and robustness. The rigorous evaluation of software tools and the implementation of FDR strategies were pivotal in bolstering the confidence of our protein identifications. Through meticulous analysis, we discerned the subtleties and strengths of various computational approaches, ensuring the accuracy of our findings and reinforcing the validity of our data. Notably, we demonstrated the feasibility of PTM analysis without the prerequisite of specific enrichment protocols. This advancement paves the way for a more streamlined and efficient exploration of PTM landscapes, revealing the multifaceted regulatory mechanisms at play within the cell. The introduction of a spheroid-specific dissociation buffer has also proved influential, enhancing the dissociation efficiency of spheroids and bolstering the robustness of our proteomic analysis. This innovation stands as a testament to the relentless pursuit of methodological refinement in SCP.

Despite these advancements, we recognize that the throughput in MS is a major bottleneck, currently capped at 120SPD. We propose that this limitation could be mitigated through the integration of nDIA with multiplexed approaches, including tandem mass tags and multiplexed DIA. This combinatorial strategy holds the potential to exponentially increase throughput and analytical depth. Meanwhile, exciting progress has been made in non-MS techniques for identifying and potentially sequencing individual proteins^38,39^. These methods draw inspiration from nucleic acid sequencing technologies and include single-molecule peptide sequencing through Edman degradation or amino peptidases in flow cells, as well as the use of nanopore sequencing adapted for proteins. While our results reported a dynamic range spanning several orders of magnitude, it is important to note that the entire dynamic range is not fully quantitative. Moreover, although our SCP workflow has shown high reproducibility and effective protein identification, other aspects such as processing volume and material of the chip could affect sensitivity and should be further investigated. Specifically, minimizing surface adsorption losses and buffer evaporation are crucial for ensuring the integrity and sensitivity of single-cell analyses. To demonstrate the universality and robustness of our Chip-Tip method, we have tested it with an Orbitrap Fusion Lumos system using HEK293T (human embryonic kidney 293T) cells, achieving approximately 3,000 protein identifications when analyzing the samples with DIA using Orbitrap detection. Moreover, another study has shown that the Chip-Tip method also performs well on the timsTOF Ultra instrument from Bruker identifying up to 4,000 proteins per single HEK293T (ref. ^40^). These results underscore the versatility of our method across various commercial devices, further validating its outstanding performance.

Our application of SCP analysis to EB induction from hiPSCs demonstrates the power of this technology for addressing biologically relevant questions. This workflow is poised to become a cornerstone in future SCP studies, illuminating the technological advancements and applications in complex dynamics of cellular function and disease. We foresee the integration of single-cell genomic, proteomic and other omics measurements as a transformative approach. We are on the verge of an era where multi-omics experiments will become the norm, and their synergistic application promises to provide a more comprehensive understanding of cellular states, particularly in disease contexts.

## Methods

### Cell lines

Different human cell lines (HeLa) were cultured in DMEM (Gibco, Invitrogen), supplemented with 10% fetal bovine serum, 100 U ml^−1^ penicillin (Invitrogen) and 100 μg ml^−1^ streptomycin (Invitrogen), at 37 °C in a humidified incubator with 5% CO_2_. Cells were collected at roughly 80% confluence by washing three times with PBS (Gibco, Life technologies). Cells were then resuspended in degassed PBS at 200 cells per µl for isolation within the cellenONE.

### Spheroid formation

Before spheroid formation, HCT116 cells were seeded to P15 plates and grown to a confluence of 70–90%. Subsequently, cells were washed with PBS (Gibco, Life Technologies) and detached from the plate with trypsin. Following this the cells were counted using a Corning Cell Counter (Sigma-Aldrich). For the multicellular spheroid generation, 7,000 cells were seeded on ultra-low attachment 96-well plates (Corning CoStar, Merck). The spheroids were cultured for 72 h at 37 °C, in a humidified incubator with 5% CO_2_. Cell medium was refreshed after 48 h, by aspirating half the old medium (making sure not to alter the spheroid) and adding the same amount of fresh medium. Subsequently the spheroids were treated for 24 h with 2 μM 5-FU (previously identified as the half-maximum inhibitory concentration (IC_50_) for this cell line). After 24 h, the spheroids were transferred to an Eppendorf tube using a p1000 and washed three times with ice cold PBS. The spheroids were disaggregated using the Dissociation Buffer for Spheroids (Cellenion SASU) by incubating for 15 min of shaking at room temperature followed by 10 min of shaking at 4 °C. The Eppendorf tube was gently shaken to induce mechanistical disintegration as well. Following this 10 μl of cell solution was diluted in 490 μl of cold PBS before sample preparation in the CellenONE.

### hiPSC culture and EB formation

hi12 (ref. ^32^) iPSCs were cultured at 37 °C and 5% CO_2_ in a humidified incubator (Thermo Fisher Scientific) on LN-521-coated dishes (BioLamina) in NutriStem medium (Sartorius). For EB induction, the cells were detached using TrypLE Express (Thermo Fisher Scientific), resuspended in NutriStem supplemented with 10 µM of Y-27632 and cultured in suspension on low adhesion flasks. After 1 week in suspension, the EBs were plated in gelatin-coated culture dish in DMEM medium (Gibco) supplemented with 20% (vol/vol) fetal bovine serum (Gibco), 2 mM l-glutamine and 1% (wt/vol) nonessential amino acids (Gibco). The plated EBs were dissociated into single-cell suspension after 1 week along with hi12 cells using TrypLE Express sorted using cellenONE for SCP analysis (described below).

### Sample preparation with Chip-Tip

Sample lysis and digestion is performed within the proteoCHIP EVO 96 inside the cellenONE (Cellenion SASU). The chip can be manufactured using either polypropylene or Teflon. This process begins with the manual deposition of 2 µl of immiscible liquid hexadecane oil into each well of the chip, which is then positioned on the target plate. The entire system is cooled to 8 °C to ensure the hexadecane oil layer solidifies effectively. Following this, a master mix consisting of 0.2% DDM (D4641-500MG, Sigma-Aldrich), 100 mM TEAB, 20 ng µl^−1^ trypsin and 10 ng µl^−1^ lys-C in a volume of 300 nl is dispensed into each well.

The cell isolation process uses the precision of the cellenONE module to sort individual cells, which are selected based on morphological criteria (diameter range of 22–30 µm and a maximum elongation factor of 1.6), into each well. The proteoCHIP EVO 96 is then subjected to a controlled incubation phase at 50 °C with 85% relative humidity for 1.5 h within the instrument’s environment. As the melting point of hexadecane is 18.2 °C, the aqueous sample solution will be covered by the hexadecane oil layer on top to prevent evaporation. This maintains constant enzyme and chemical concentrations, and the oil layer thereby contributes to ensure reproducible processing efficiency. After incubation, the system’s temperature is reduced to 20 °C to stabilize the conditions postreaction.

On completion of the incubation, the proteoCHIP EVO 96 is taken out of the cellenONE and processed further. This involves the manual addition of 4 µl of 0.1% formic acid to each well, followed by a chilling period at 4 °C to refreeze the oil. In parallel, the Evotips (Evosep Biosystems) are prepared in line with the vendor’s guidelines, which include a series of rinsing, conditioning and equilibrating steps with specified solvents to prepare them for sample uptake. Afterward, another 15 µl of 0.1% formic acid is introduced to each Evotip, and the proteoCHIP is promptly inverted onto the Evotips and centrifuged at 800*g* for 20 s at 4 °C.

Once the proteoCHIP EVO 96 is removed, the Evotips undergo a customized procedure that deviates slightly from the standard vendor’s protocol. The samples are first loaded onto the Evotips by centrifugation at 800*g* for 60 s, ensuring that the peptides are fully captured by the tip matrix. After loading, the Evotips are meticulously washed with 20 µl of Solvent A, and the centrifugation step is repeated for another 60 s at 800*g* to remove any nonspecifically bound substances, thereby increasing the purity of the captured peptides. The final step involves adding 100 µl of Solvent A to the Evotips and spinning them for a brief 10 s at 800*g*. The samples are then ready for LC–MS/MS analysis.

### Sample preparation for the 96-well plate workflow

Using a standard 96-well plate (Eppendorf twin.tec PCR Plate 96 LoBind), HeLa cells were diluted with degassed PBS to 100–200 cells per μl. The 96-well plate was placed inside the CellenONE, where 1 µl of master mix buffer was automatically injected into each well of the chip and then positioned on the target plate. To determine the suitability for SCP analysis, 5–10 µl of cells were picked up by the CellenONE via a standard autosampler to assess morphology, density and diameter, targeting cells with a diameter of 15–35 µm. If the cells met the criteria, the CellenONE module sorted individual cells based on a diameter range and an elongation factor not larger than 1.8 into the wells. After sorting, 500 nl of master mix buffer was injected into each well by CellenONE via a standard autosampler. The 96-well plate was then subjected to a controlled incubation phase at 50 °C with 85% relative humidity for 1 h within the instrument environment, involving an automatic cycle system that added 500 nl of water to each well until the process was complete. After incubation, the temperature was reduced to 20 °C to stabilize the conditions postreaction. On completion, the 96-well plate was removed from the CellenONE and processed further, with 3.5 µl of 0.1% TFA/1% DMSO manually added to the wells. The plates were then sealed with matching 96-well plate covers and placed in the Vanquish Neo, allowing for the direct injection of 4 µl ready for MS analysis.

### LC–MS/MS

The LC–MS/MS analysis was conducted using an Orbitrap Astral mass spectrometer (Thermo Fisher Scientific) coupled with an Evosep One chromatography system (Evosep Biosystems). The sample runs were set up for a 40SPD, 80SPD or 120SPD Whisper protocol. We used Aurora Elite TS analytical columns (15 cm × 75 µM, IonOpticks) with an EASY-Spray ion source (Thermo Fisher Scientific) for the 40SPD method. A rapid column (5 cm × 75 µM, IonOpticks) was used for the 80SPD and 120SPD methods with a Nanospray Flex ion source (Thermo Fisher Scientific). The Orbitrap Astral operated with a resolution setting of 240,000 for full MS scans across a mass-to-charge range of 380 to 980 *m/z*. The automatic gain control (AGC) for full MS was adjusted to 500%. MS/MS scans employed various nDIA methods with isolation windows and ion injection times tailored to the specificity of the analysis—these included settings such as 2Th3ms, 4Th6ms, 8Th12ms, and 16Th24ms. The MS/MS scanning spanned the same *m/z* range of 380 to 980. Fragmentation of the isolated ions was carried out using higher-energy collisional dissociation set at a normalized collision energy of 27%. For the 40SPD gradient, single-cell samples were distributed among the different nDIA methods: 12 for 2Th3ms, 12 for 4Th6ms, 24 for 8Th12ms and 12 for 16Th24ms. Samples composed of 10, 20 and 40 cells were analyzed in sets of four using the 2Th3ms, 4Th6ms and 8Th12ms methods, respectively.

For the analysis with Orbitrap Astral coupled with Vanquish Neo LC, the sample runs were configured for 60 SPD, with each run having a total gradient of 14 min and not exceeding 10 min for equilibrating and loading using Vanquish Neo. The analysis used Aurora Elite TS analytical columns and was interfaced online using an EASY-Spray source. Additionally, FAIMS Pro Duo was employed to reduce background interference, enhancing the identification sensitivity of low-abundance peptides. The detailed MS parameters were as follows: the ion source parameter had a spray voltage of 1.9 kV and a capillary temperature of 270 °C, with FAIMS compensation voltage set to −48 V. For Orbitrap MS full scans, the resolution was 240,000, the normalized AGC target was 500%, the maximum injection time was 100 ms, the RF lens was 45% and the scan range was 400–800 *m/z*. For Astral DIA MS2 scans, the precursor mass range was 400–800 *m/z*, the DIA window type was set to Auto with window placement optimization on, the DIA window mode was *m/z* range, the higher-energy collisional dissociation normalized collision energy was 25%, the scan range was 150–2,000 *m/z*, the RF lens was 45%, the normalized AGC target was 800% and the loop control time was 0.6 s. The DIA isolation window was 20Th and the maximum injection time was 40 ms.

### Data analysis

For the analysis using Spectronaut v.18 (Biognosys), raw files underwent a library-free directDIA+ approach, employing the human reference database from the UniProt 2022 release, which contains 20,588 sequences, alongside an additional 246 common contaminant sequences. Notably, cysteine carbamidomethylation was not included as a modification, while variable modifications were set for methionine oxidation and protein N-terminal acetylation. The precursor filtering was based on the *Q* value, and cross-run normalization was not applied. For the phosphorylation search, phosphorylation on serine, threonine and tyrosine was set as a variable modification.

For the phosphorylation analysis, a filter of PTM.SiteProbability ≥0.75 was applied to ensure confident identification and site localization. For the glycan oxonium ion analysis, the rawrr package was used to extract a mass list of 163.060, 204.087, 274.092, 366.139, 485.046 and 657.140 from the single-cell raw file, representing different glycan structures. A mass tolerance of 5 ppm was set for the XIC.

In the case of DIA-NN (v.1.9) searches, the raw data files were first converted to .mzML format. The analyses were then conducted in a library-free mode with some modifications: the maximum number of variable modifications was limited to two, and as with Spectronaut, cysteine carbamidomethylation was not set as a modification. It was configured to use highly heuristic protein grouping, and the MBR feature was activated. Results from both the first pass search and the MBR search are shown in Fig. 2a,b.

For the analysis in Fig. 2c, a total of 24 single cells were analyzed using the 4Th6ms method. Thirteen searches were conducted in total: the first search included the initial two single-cell samples, the second search included the first four single-cell samples, and this pattern continued until the 12th search, which included all 24 single-cell samples. The 13th search combined all 24 single-cell samples with additional 20-cell samples using the 4Th6ms method. The figure displays the identifications from the first four cells.

For the entrapment analysis, entrapment peptides were processed using an updated version of a tool known as ‘mimic’ from the percolator repository. This tool shuffles the target database ninefold and appends the shuffled proteins flagged as mimic and ‘Random_XXXX’, preserving the original amino acid composition from the fasta file. This strategy ensures a rigorous assessment of the FDRs in our proteomic analysis. The entrapment analysis utilized a database containing both the human reference database and its 1× mimic database. The analysis was conducted using both Spectronaut and DIA-NN with the same settings as previously mentioned. The results from the first pass search in the DIA-NN analysis were shown. All the analysis log files and settings files were summarized in Supplementary Data 1.

For the analysis in Fig. 5, the data was analyzed using Spectronaut (v.18). The differential analysis was calculated using the Spectronaut comparison. Consequently, R was used for further analysis of the data. The geneset enrichment was calculated using the gseGO function from the Clusterprofiler package. For visualization of the data the enrichplot package was used. Pathview, treeplot and UpSet plot were used to create the associated figures. For the creation of the heatmap, the heatplot function from the enrichplot package was used.

For the analysis in Fig. 6, missing values were imputed as random draws from the low quantile values (2.5th percentile) of the data distribution using the impute.pa function of the imp4p package v.1.2, with cells belonging to EBs and iPSCs set as the two conditions.

### Statistics and reproducibility

No statistical method was used to predetermine sample size. For single-cell analysis, samples corresponding to more than one cell due to technical error, which was highlighted by the CellenONE sorter were excluded from analysis since the analysis only aimed to study single cells. The experiments were not randomized. The Investigators were not blinded to allocation during experiments and outcome assessment.

### Reporting summary

Further information on research design is available in the Nature Portfolio Reporting Summary linked to this article.

## Online content

Any methods, additional references, Nature Portfolio reporting summaries, source data, extended data, supplementary information, acknowledgements, peer review information; details of author contributions and competing interests; and statements of data and code availability are available at 10.1038/s41592-024-02558-2.

## Supplementary information

Reporting Summary

Supplementary Table 1 Identified proteins and peptides from HeLa single cells using the Chip-Tip method.

Supplementary Table 2 Protein quantities in iPSCs and EBs single-cell samples.

Supplementary Data 1 Analysis log files and settings files from Spectronaut and DIA-NN.

## Source data

Source Data Figs. 1–4 and 6 and Extended Data Fig. 7 Statistical source data.

## Data Availability

The MS proteomics data have been deposited to ProteomeXchange Consortium (http://proteomecentral.proteomexchange.org) via the PRIDE partner repository with dataset identifier PXD049211 and PXD049181 (hiPSC and EB datasets) and the iProX partner repository with the dataset identifier PXD054944 (SCP samples with the Vanquish Neo-FAIMS workflow). Source data are provided with this paper.
