# Supplementary material for: Enhanced sensitivity and scalability with a Chip-Tip workflow enables deep single-cell proteomics
Source: Nat Methods. 2025 Jan 16;22(3):499–509. doi: 10.1038/s41592-024-02558-2 (PMC11903336; doi:10.1038/s41592-024-02558-2)
Supplement: Supplementary file 1 — Reporting Summary [file 41592_2024_2558_MOESM1_ESM.pdf]

nature portfolio

Corresponding author(s): Jesper Velgard Olsen

Last updated by author(s): Aug 21, 2024

Reporting Summary

Nature Portfolio wishes to improve the reproducibility of the work that we publish. This form provides structure for consistency and transparency in reporting. For further information on Nature Portfolio policies, see our Editorial Policies and the Editorial Policy Checklist.

Statistics

For all statistical analyses, confirm that the following items are present in the figure legend, table legend, main text, or Methods section.

n/a	Confirmed	
☐	☒	The exact sample size (*n*) for each experimental group/condition, given as a discrete number and unit of measurement
☐	☒	A statement on whether measurements were taken from distinct samples or whether the same sample was measured repeatedly
☐	☒	The statistical test(s) used AND whether they are one- or two-sided<br>*Only common tests should be described solely by name; describe more complex techniques in the Methods section.*
☐	☒	A description of all covariates tested
☐	☒	A description of any assumptions or corrections, such as tests of normality and adjustment for multiple comparisons
☐	☒	A full description of the statistical parameters including central tendency (e.g. means) or other basic estimates (e.g. regression coefficient) AND variation (e.g. standard deviation) or associated estimates of uncertainty (e.g. confidence intervals)
☐	☒	For null hypothesis testing, the test statistic (e.g. *F*, *t*, *r*) with confidence intervals, effect sizes, degrees of freedom and *P* value noted<br>*Give P values as exact values whenever suitable.*
☒	☐	For Bayesian analysis, information on the choice of priors and Markov chain Monte Carlo settings
☐	☒	For hierarchical and complex designs, identification of the appropriate level for tests and full reporting of outcomes
☒	☐	Estimates of effect sizes (e.g. Cohen's *d*, Pearson's *r*), indicating how they were calculated

Our web collection on statistics for biologists contains articles on many of the points above.

Software and code

Policy information about availability of computer code

Data collection	Mass spectrometry data was acquired using the Orbitrap Astral MS, using Thermo Tune software (version: 0.4 or higher)
Data analysis	Proteomics data was analysed using Spectronaut (v18 and v19).<br>DIA-NN version 1.9.<br>ClusterProfiler v4.8.3.<br>imp4p version 1.2.

For manuscripts utilizing custom algorithms or software that are central to the research but not yet described in published literature, software must be made available to editors and reviewers. We strongly encourage code deposition in a community repository (e.g. GitHub). See the Nature Portfolio guidelines for submitting code & software for further information.

Data

Policy information about availability of data

All manuscripts must include a data availability statement. This statement should provide the following information, where applicable:

- Accession codes, unique identifiers, or web links for publicly available datasets
- A description of any restrictions on data availability
- For clinical datasets or third party data, please ensure that the statement adheres to our policy

The databases used for data analysis consisted of the Uniprot 2022 release, which contains 20,588 sequences, alongside an additional 246 common contaminant sequences.

The mass spectrometry proteomics data have been deposited to ProteomeXchange Consortium (http://proteomecentral.proteomexchange.org) via the PRIDE partner repository with dataset identifier PXD049211 and PXD049181 (hiPSC and EB datasets) and the iProX partner repository with the dataset identifier PXD054944 (SCP samples with the Vanquish Neo-FAIMS workflow). These datasets are publicly available.

Research involving human participants, their data, or biological material

Policy information about studies with human participants or human data. See also policy information about sex, gender (identity/presentation), and sexual orientation and race, ethnicity and racism.

Reporting on sex and gender	Data reported in this work does not involve human participants.
Reporting on race, ethnicity, or other socially relevant groupings	Data reported in this work does not involve human participants.
Population characteristics	Data reported in this work does not involve human participants.
Recruitment	Data reported in this work does not involve human participants.
Ethics oversight	Data reported in this work does not involve human participants.

Note that full information on the approval of the study protocol must also be provided in the manuscript.

Field-specific reporting

Please select the one below that is the best fit for your research. If you are not sure, read the appropriate sections before making your selection.

☒ Life sciences ☐ Behavioural & social sciences ☐ Ecological, evolutionary & environmental sciences

For a reference copy of the document with all sections, see nature.com/documents/nr-reporting-summary-flat.pdf

Life sciences study design

All studies must disclose on these points even when the disclosure is negative.

Sample size	No sample size calculation was performed, the number of samples used per experiment were chosen based on previous experience to obtain enough statistical power in DIA experiments.
Data exclusions	For single-cell analysis, samples corresponding to more than one cell due to technical error, which was highlighted by the CellenONE sorter were excluded from analysis since the analysis only aimed to study single cells.
Replication	To assess technical variability of the mass spectrometry measurements each experiment was performed using at least three injection replicates for the method evaluation part. For other single-cell analysis at least 10 cells were analyzed per conditions.<br>All attempts at replicating the data were successful.
Randomization	No randomization was performed for technical benchmark.
Blinding	Researchers were not blinded to perform the experiments reported in this work.<br>This was required as to keep a strict overview of mass spectrometry method selection.

Reporting for specific materials, systems and methods

We require information from authors about some types of materials, experimental systems and methods used in many studies. Here, indicate whether each material, system or method listed is relevant to your study. If you are not sure if a list item applies to your research, read the appropriate section before selecting a response.

Materials & experimental systems

n/a	Involved in the study
☒	☐ Antibodies
☐	☒ Eukaryotic cell lines
☒	☐ Palaeontology and archaeology
☒	☐ Animals and other organisms
☒	☐ Clinical data
☒	☐ Dual use research of concern
☒	☐ Plants

Methods

n/a	Involved in the study
☒	☐ ChIP-seq
☒	☐ Flow cytometry
☒	☐ MRI-based neuroimaging

Eukaryotic cell lines

Policy information about cell lines and Sex and Gender in Research

Cell line source(s)	HeLa cells were obtained from ATCC. hiPSCs hi12 were derived from hFF provided by ATCC.
Authentication	The cell lines used were not authenticated.
Mycoplasma contamination	All cell lines tested negative for mycoplasma contamination.
Commonly misidentified lines (See ICLAC register)	This study does not involve commonly misidentified cell lines.
